# Pain Relief Interventions in Australian Livestock Husbandry: A Review of Animal Welfare and Pain Duration

**DOI:** 10.3390/ani14131901

**Published:** 2024-06-27

**Authors:** Lee Metcalf, Sabrina Lomax, Dominique Van der Saag, Sanjay Garg, Peter J. White

**Affiliations:** 1 School of Veterinary Science, University of Sydney, Camperdown, NSW 2006, Australia; dominique.van.der.saag@sydney.edu.au (D.V.d.S.); p.white@sydney.edu.au (P.J.W.); 2 School of Life and Environmental Sciences, University of Sydney, Camperdown, NSW 2006, Australia; sabrina.lomax@sydney.edu.au; 3 UniSA Clinical & Health Sciences, University of South Australia, Adelaide, SA 5001, Australia; sanjay.garg@unisa.edu.au

**Keywords:** pain, sheep, cattle, livestock, analgesia, production animals

## Abstract

**Simple Summary:**

It is well established that animals feel pain akin to humans, although the expression of that pain is not as easy to perceive, especially considering that many species actively conceal or disguise pain, distress, or weakness. Current methods of husbandry practices used to improve welfare or production cause inherently painful tissue damage. Current interventions focus on immediate pain relief, but research indicates persistent pain behaviours post procedure, with pain experienced after routine husbandry procedures such as castration, tail docking, dehorning, and mulesing reported as lasting for days and sometimes weeks after the operation, affecting the animal’s welfare and production performance. As livestock handlers, animal owners and veterinarians become better at recognising situations where pain and distress are experienced, efforts are increasing to improve pain mitigation methods. The challenges of avoiding multiple handling of livestock, or relying on owner compliance, may be found in developing long-acting pain relief solutions.

**Abstract:**

In veterinary medicine and livestock production, ensuring good animal husbandry is vital for the physical and emotional wellbeing of animals under our care. Pain poses challenges for assessment and mitigation, especially in species unable to express pain overtly. This review examines current pain mitigation interventions in routine husbandry, focuses on the duration of pain after procedures and implications for animal welfare. Pain behaviours have been observed for days or weeks after regular husbandry procedures, and many studies have noted pain-related behaviour persisting until study finalisation, suggesting potential undocumented pain beyond study completion. Current products registered in Australia for pain mitigation in livestock primarily target immediate pain associated with procedures. The future of pain relief in livestock demands longer-acting solutions to address post-procedural pain adequately. Providing pain relief for at least 72 h post surgery is recommended, but current products require retreatment intervals to achieve this, posing practical challenges, especially in livestock. Methods of pain relief provision, such as voluntary consumption of medicated feed, transdermal medication delivery and long-acting formulations offer potential solutions for prolonged pain relief, with research ongoing in these areas. There is a need for further research and development of longer-acting pain relief to ensure optimal welfare of livestock.

## 1. Introduction

Within veterinary medicine and livestock production, it is recognised that good animal husbandry is necessary to ensure the physical and emotional well-being of livestock and companion animals. The concept of “a life worth living” [1] places the responsibility on animal owners and veterinarians to ensure that there is a balance between positive and negative experiences in an animal’s lifetime; that “suffering is somehow compensated for by pleasant experiences”. There are some invasive husbandry procedures performed on livestock that are painful but considered necessary, either to ensure ongoing welfare for the animal or to facilitate efficient and safe management. These procedures result in physical injury to tissue, and in Australian livestock are often performed without any pre- or post-procedural anaesthesia or analgesia, although this is changing due to some Australian States having legislative requirements for pain relief for certain procedures or at certain ages [2,3]. Even in companion animals, owners are often provided with the choice as to whether post-surgical pain relief is provided, and the decision is frequently driven by cost or owner perception of animal pain [4].

Pain is an “aversive sensation and feeling associated with actual or potential tissue damage, or described in terms of such damage” [5]. There are different types of pain, which may be classified by the duration, the part of the body in which it is located, symptoms, syndromes, or mechanisms [6]. The importance and challenges of pain assessment in animals have been covered extensively in previous research [7,8,9,10,11]. Understanding the pathways and timing of the pain experience is necessary to determine the best method of pain mitigation in any species, whether or not they can express that pain.

It is interesting to note that in the early days of veterinary anaesthetics, anaesthesia was originally used for restraint rather than pain relief. The drugs initially used would induce paralysis but not necessarily provide any pain relief either during or after a procedure [12]. Some veterinarians and producers misunderstood that general anaesthesia or heavy sedation which chemically restrained the animal was not synonymous with analgesia and assumed that a lack of reaction to pain during and after surgery was due to an analgesic effect of the anaesthesia [8,13].

Practices such as branding and ear tagging/notching of livestock for identification purposes; dehorning and disbudding cattle and goats to prevent injury to other members of the herd, or handlers; castration and spaying of livestock and companion animals to prevent unwanted pregnancies and aggressive mating behaviour; and tail docking and mulesing of sheep to prevent fly strike, are all performed in Australia to improve management and production, and to ensure the holistic welfare of the animal. As an awareness of animal welfare expands, common practices are being assessed for necessity and alternatives, as well as the need for pain relief. Currently, less painful alternatives being investigated are either not viable and/or effective, or their integration may take several years or generations of breeding. It is, therefore, incumbent upon animal owners, producers, and veterinarians to ensure that animal welfare is maintained through the delivery of appropriate pain mitigation.

For production animals, consumers are increasingly demanding products such as wool, meat, milk and eggs that have been produced under proven welfare standards, including adequate pain relief [14]. There are still barriers towards the provision of pain mitigation on-farm, including cost, recognition of pain by producers, withdrawal periods, and entrenched generational farming practices [15]. Recent surveys of sheep and cattle producers have shown that only one-quarter of those surveyed are providing pain relief for routine procedures, with the most common objections from those producers not using pain relief being the time it takes, or that they do not believe it is necessary [16,17].

Over the last 27 years, the Five Domains Model for animal welfare assessment has been developed and updated to provide a way to evaluate the welfare of individuals or groups of animals [18] with particular emphasis on well-being and positive experience. The Five Domains indicate that the welfare of animals is associated with both mental and physical aspects and infers that animals should be provided with adequate nutrition, environment, the ability to behave naturally and receive adequate healthcare whilst ensuring that the animal’s mental state is also protected. Good animal husbandry is necessary to ensure the health and wellbeing of livestock and the reality exists that some invasive husbandry procedures conducted for welfare or management purposes are painful. Studies in livestock have shown that the response of animals to pain is influenced by several different parameters, such as sex, age, body weight, prior experience and familiarity with the environment [19]. The emotional distress experienced during aversive procedures has also been demonstrated in livestock, with studies in calves showing an aversion to the location of a painful experience such as hot-iron disbudding [20] or preference for analgesia [21], and sheep displaying handler aversion for several months after a mulesing operation [22]. An important component of animal welfare, therefore, is appropriate pain relief before, during and after painful procedures, to ensure that the human-animal interaction is as stress-free as possible and that the restriction of behavioural interactions and negative experiences are minimized [23,24,25].

There has been a historical assumption [13,15] that neonates have less developed pain perception than older animals, and therefore procedures should be conducted as soon as possible after birth—this has even been the case with human neonates, even though the physiological markers of pain in humans are measurable from 26 weeks gestation [26]. Studies in lambs have shown that reaction to pain changes over time from birth, with one study showing an increase in electroencephalographic (EEG) response to castration as lambs increased in age from 1 day to 6 weeks [27]. A study of EEG responses of piglets that were tail-docked at either 2 or 20 days of age showed that the procedure appeared less acutely painful when performed soon after birth rather than at 20 days of age [28]. In contrast, a study of lambs [29] found that those animals castrated within a day of birth when compared with those castrated at 10 days of age, showed a higher pain response when tail docked at 3–5 weeks of age, leading to the conclusion that a “noxious stimulus” early in life (such as the pain associated with castration) can cause increased pain sensitivity later in life. This concept was further demonstrated in dairy calves disbudded at 3 days of age vs. 35 days of age [30]. The experience of pain has even been shown to be intergenerational—a study in sheep [31] found that ewes experiencing pain from tail docking or a simulated lipopolysaccharide (LPS) infection (*E.coli* LPS challenge) at 3–4 days of life showed higher levels of pain-related behaviour as adults when lambing. In addition, the LPS-treated ewes gave birth to lambs who also displayed a lower tolerance to pain at 2–3 days of age. There may therefore be a long-term and even trans-generational effect of pain experienced in neonates which would be worth further examination.

There is a lack of clarity and consistency for those in the industry when considering the legislative requirements for pain relief for livestock throughout the different states in Australia. The legal requirement for pain relief in mulesing of sheep, for example, ranges from support for the voluntary adoption of pain relief by the NSW government, with some technical assistance to find alternatives to mulesing [32], to the Victorian government’s Prevention of Cruelty to Animals Regulations 2019 making the performance of mulesing without pain relief an offence [2]. Some industry bodies (for example, some wool buying groups) have taken a lead with regards to animal welfare, with certain requirements of their producers [33,34]; however, as up to 75% of the country’s cattle and sheep farmers are not voluntarily providing pain relief for other routine procedures [16,17], it seems that until there is clear nationwide legislation with specific requirements, pain relief in the production space will remain inconsistent and often inadequate.

This review examines the current pain mitigation interventions in routine husbandry practice; of particular concern for animal welfare is the duration of pain following routine procedures, which highlights the need for more effective pain management strategies. This review examines the existing landscape, identifies gaps in available pain relief, and proposes avenues for future research to ensure the best pain relief and welfare standards in husbandry practices.

## 2. Search Methodology

A literature review was conducted by a search of CAB Abstracts via Web of Science (1910—present) and BIOSIS Previews via Web of Science (1926—present). Further databases were not included due to frequent overlap of articles across databases. Keywords included “pain”, “chronic pain”, “pain relief”, “husbandry”, “welfare”,” long acting”, “extended release”, “sustained release”.

To specifically address pain duration after husbandry, a search was conducted using PICO methodology:
**PICO Elements****Search Terms****Boolean Operator**Patient/populationCattle Sheep Pigs Goatscattle OR cow* OR bovine* OR steer OR sheep OR ovine OR ram OR wether OR goat* OR kid* OR caprine OR lamb* OR pig* OR sow* OR porcine -InterventionCastration Spay Mulesing Dehorning/disbudding Tail dockingCastrat* OR spay* OR spey* OR tail* OR mule* OR *horn* OR *bud*ANDComparison---OutcomeLong term painPain* AND long* NEAR termAND(*) is a truncation symbol to search for all endings to a word.

In total, 150 articles were reviewed for inclusion and then some were excluded for the following reasons:

1. Studies that tracked pain up to 36 h only, as the currently available pain mitigation products provide relief for up to 36 h;
2. Studies that were for surgical procedures not considered as routine husbandry (such as orthopaedic surgery);
3. Articles that were not in English;
4. Articles that were reviews rather than original studies.

A small selection of hand-picked information found using the standard literature review search method was also included, resulting in a total of 33 studies included in the review and presented in Table 1.

To expressly search for current pain mitigation, a review of two veterinary drug handbooks [35,36] was conducted to identify common analgesic and anti-inflammatory drug classes (Table 2). Personal knowledge of the authors was used to identify common off-label use in Australian Practice.

To identify those products registered specifically for post-surgical pain relief in livestock, a search was conducted of the Public Chemical Registration Information System (PubCRIS) database of the Australian Pesticides and Veterinary Medicines Authority (APVMA) [37]. The search terms used were the active constituents of interest, based on the results of Table 2. Each search result (“Product List”) based on the active constituent was exported as a CSV file to Microsoft Excel, and the registered host/pest and claim identified and sorted alphabetically. Where several identical products were found (generics), the label of the first registered product (based on registration date) was reviewed.

Products included for review were then identified based on the host alias of “beef”, “beef calf”, “bos indicus”, “bos taurus”, “bovine”, “buffalo”, “bull”, “bullock”, “calf”, “cow”, “heifer”, “steer”, “lamb”, “sheep”, “ewe”, “ram”, “swine”, “pig”, “gilt”, “sow”, “weaner” and “wether”, and the pest alias of “inflammation” and “pain”. The labels of those included products were reviewed on PubCRIS to identify the relevant particulars (Table 3). Products which provide general anaesthesia were not included, as these are not used in the context of routine on-farm husbandry in Australia.

**Table 1:** Summary of studies showing pain timeframes post routine husbandry.

Species, Breed	Age	Procedure, Method	No. of Animals (Per Group)	Acute Pain Relief Received *	Study Duration (Days)	Duration of Pain (Days)	Parameters Measured as Indicator of Pain	Parameters Indicating Long Term Pain **	Ref
Goat, Saanen	9–14 days	Disbudding, cautery vs. caustic paste vs. liquid nitrogen vs. clove oil injection vs. sham	50 (10)	None	42	42	Cortisol, haptoglobin up to 24 h. Skin surface temperature, Average Daily Gain (ADG), lesion measurements, lying time, head and body shaking, head scratching, self grooming, feeding	Prolonged healing (note behaviour was tracked for 24 h, ADG for 7 days).	[38,39]
Cattle, Holstein or Jersey	24–38 days	Disbudding, cautery	24 (13 test and 11 control)	Local anaesthetic (lignocaine block) vs. placebo (saline), all received meloxicam 1 mg/kg per os (PO)	11	11	Ethogram of behaviour (head scratch, tub, shake, ear flick, tail flick, buck/jump, grooming, transition to lying)	Behavioural—head shake and ear flick	[40]
Cattle, Holstein or Jersey	3 days or 35 days	Disbudding, cautery vs. sham handling	48 (12)	Local anaesthetic (lignocaine block) for all animals, meloxicam 1 mg/kg PO for disbudded calves only	63	63	Pressure (algometer), infrared thermography, ADG, wound healing	Wound healing, pressure/pain sensitivity	[30]
Cattle, Holstein or Jersey	21–28 days	Disbudding, cautery vs. sham handling	44(11)	Lignocaine block vs. placebo (saline), then meloxicam 1 mg/kg PO for disbudded calves	21	21	Pressure algometry, behaviour indicative of conditioning either 6 h or 20 days post surgery, testing a preference for analgesia	Pressure algometry, behaviour indicative of conditioning showing a preference for analgesia	[21]
Cattle, Holstein	7 days and 28 days	Disbudding, cautery vs. sham handling	30 (10)	Perineural 2% lignocaine, meloxicam 0.5 mg/kg intravenous (IV)	105	105	Visual analogue scale, quantitative sensory testing (pressure-pain threshold), mechanical allodynia, withdrawal reflexes	Behavioural signs, trigeminal hyperalgesia and allodynia	[41]
Cattle, Holstein-Friesian	4–5.5 weeks	Disbudding, cautery vs. sham handling	46 (6–8)	Placebo (saline cornual injection) vs. lignocaine 2% cornual nerve block vs. lignocaine 2% cornual nerve block with meloxicam 0.5 mg/kg IV	3 (75 h)	3 (75 h)	Play behaviour, wound sensitivity via von Frey monofilaments	Wound sensitivity via von Frey monofilaments	[42]
Cattle, Holstein	16–20 weeks	Dehorning, scoop with thermocautery	12 (6)	Saline vs. meloxicam 0.5 mg/kg IV	10	10	Cortisol, substance P, activity and behaviour, heart rate, ADG	ADG	[43]
Cattle, Angus or Hereford	Newborn or weaning (214 days)	Castration, surgical	62 (15–16)	Nil vs. meloxicam 1 mg/kg PO	>300 days	7	Activity via accelerometer (7 days), ADG	Activity via accelerometer (for 7 days)	[44]
Cattle, Hereford X or Black Angus	37–59 days	Castration, surgical vs. sham handling	158 (52–54)	Placebo (saline intramuscular (IM) injection) vs. Meloxicam 0.5 mg/kg IM vs. no injection for sham animals	14	14	Hair cortisol concentration (HCC), lying time, ADG	HCC	[45]
Cattle, Holstein	166 ± 0.4 days	Castration, surgical vs. positive control (previous castrates (steers)) vs. negative control (left intact (bulls)).	132 (44)	Flunixin 3 mg/kg IM at 0 and 48 h	126	10	Physical activity measured by pedometer, meal size and duration, lying time	Lying time (5 days), feed intake (10 days) and physical activity (10 days)	[46]
Cattle, Angus cross	2 months	Castration, surgical vs. band vs. sham handling	132 (24)	Placebo (lactated ringers solution) injected subcutaneously (SC) vs. meloxicam 0.5 mg/kg SCn	62	62	ADG and bodyweight (BW), pressure on wound, scrotal temperature, wound swelling, wound healing, behaviour: suckling, lying, standing, walking, head turn, lesion licking, foot stamp, tail flick, proximity to dam	BW and ADG, pressure on wound, wound swelling, proximity to dam	[47]
Cattle, Angus or Angus x	1 week vs. 2 months vs. 4 months	Castration, surgical vs. band vs. sham handling	108 (11–12)	None stated	69	35	Salivary cortisol and HCC, Substance P and Haptoglobin, wound temperature and healing, weigh gain, body temperature, pain behaviour, lying time, stride length	ADG at weaning (surgical castration), swelling (band castration)	[48]
Cattle, Angus x	7–8 days	Castration, surgical vs. band vs. sham handling	72 (12)	Placebo lactated Ringer’s solution injection SC vs. meloxicam 0.5 mg/mL SC	56	56	HCC, haptoglobin, serum amyloid A, scrotal swelling, scrotal temperature, wound healing, stride length, behaviour, body weight, body temperature	Inflammation (banded group), HCC	[49]
Cattle, Angus	Not stated—BW ~300 kg.	Castration, surgical	48 (12)	Placebo ring block of lactated Ringer’s solution vs. lidocaine 2% + epinephrine ring block vs. meloxicam 0.5 mg/kg SC and placebo ring block vs. meloxicam 0.5 mg/kg SC and lidocaine 2% + epinephrine ring block	28	3	Salivary cortisol, haptoglobin, scrotal temperature, stride length, visual analogue score.	Haptoglobin	[50]
Cattle, Angus x Hereford	25 ± 2 days	Castration, surgical	48 (24)	Placebo (saline) IV vs. flunixin 1.1 mg/kg IV. Lignocaine ring block (3 mL) used on all animals.	63	21–35	Healing and inflammation, wound surface temperature, ADG, Substance P, Lying behaviour	Inflammation (peaked at day 3), healing score	[51]
Cattle, Ayshire	5–7 days	Castration, surgical vs. rubber ring vs. Burdizzo vs. combination Burdizzo and ring vs. control (no castration)	40 (8)	None stated	51	42	Plasma cortisol, behaviour, lesion score	Rubber ring group showed wound directed behaviours, abnormal standing, high lesion scores	[52]
Cattle, Holstein	28 days	Castration, surgical vs. rubber ring	21 (10 and 11)	Lignocaine 2% local anaesthetic and meloxicam 0.5 mg/mL SC for all calves	56	56	Wound healing, inflammation, weight gain, feed intake, lying time, wound-directed behaviours	Rubber ring group showed lower weight gain after rubber ringing, scrotal inflammation, wound-directed behaviours, reduced lying	[53]
Cattle, Holstein	4–5 months	Castration, surgical vs. rubber ring	60 (15)	Placebo (saline) vs. meloxicam 1 mg/kg PO	3	3	Substance P, heart rate, cortisol, lying time, tail movements, observed painful behaviour, swelling (inflammation)	Lying time, observed painful behaviour, swelling	[54]
Cattle, Simmental or Simmental x Red Holstein	21–28 days	Castration, rubber ring vs. Burdizzo vs. sham handling	70 (10–15)	Placebo (saline) local infiltration 10 mL vs. lignocaine 2% local infiltration 10 mL	90	90	Expression of pain during castration, serum cortisol (to 72 h), behaviour, posture, scrotal condition including palpation, histology	Reaction to local palpation (up to 50 days), abnormal standing (up to 90 days) (rubber ring group)	[55]
Cattle, Holstein	Adult (lactating)	Tail docking, rubber ring vs. control	64 (16)	None vs. caudal epidural anaesthetic lignocaine 2% 4 mL	6	6	Tail movement and position, posture, milk production, feed intake	Tail movement and position, posture	[56]
Cattle, Holstein	12 months	Tail docking, rubber ring vs. undocked control	164 (133 + 31 control)	None stated	Tested at 6.2 ± 1.9 years of age after docking <12 months old	Tested at 6.2 ± 1.9 years of age	Pressure, thermal and pinprick sensitivity tests	Pressure, heat and cold sensitivity, pinprick sensitivity test	[57]
Pig, not stated	9 or 17 weeks	Tail docking, surgical—2/3rd removed vs. 1/3rd removed vs. sham handling	108 (12–23)	None	112	56	Mechanical Nociceptive Thresholds (MNT)	Mechanical Nociceptive Thresholds (MNT)	[58]
Pig, Landrace x Large white	2 days	Tail docking, clip vs. cautery vs. control	120 (40)	None	21 weeks	N/A	Histology of tail at slaughter	Histology showing evidence of neuroma formation indicative of neuropathic pain.	[59]
Pig, Landrace/Large White x synthetic sire line	3 days	Tail docking, cautery	16 (4)	None	112	112	Examination of tail stump at 1, 4, 8 and 16 weeks post amputation for histopathological changes (healing, neuroma formation	Traumatic neuromata after 28 days and ongoing past 16 weeks (112 days).	[60]
Pig, Landrace/Large White x synthetic sire line	3 or 63 days	Tail docking, amputation vs. sham handling	96 (8)	3 days old: none. 63 days old: meloxicam 0.2 mg/kg IM	112	112	Examination at 1, 8 and 16 weeks for changes in gene expression, traumatic neuroma development and inflammation	Changes in gene expression associated with both inflammatory pain and neuropathic pain	[61]
Pig, Piétrain x Hypor	2–8 days	Castration, surgical	186 (95 and 91)	CO_2_ anaesthesia vs. none	8	6	Behaviour: general (suckling, socialisation, movement, suckling) specific pain related (huddling, trembling, spasms, scratching, tail wagging), posture, isolation	Pain-related behaviours, walking frequency, lying, sucking, interaction	[62]
Sheep, White Swiss Mountain	>10 to 24 weeks	Castration, surgical vs. Burdizzo vs. rubber ring vs. sham handling	70 (10)	Lidocaine 2% 4 mg/kg infiltration injection vs. bupivacaine 0.5% 1.5 mg/kg infiltration injection	30	21	Response to pain during castration, cortisol levels up to 48 h, food intake day of castration, behaviours and postures, lesions, palpation, bodyweight measurements, histology	Local palpation, average daily gain	[63]
Sheep, White Swiss Mountain and x Charolais	2–7 days	Castration, ring vs. Burdizzo vs. sham handling	70 (11–12)	Placebo (saline) vs. lidocaine 4 mg/kg infiltration	90	21	Response to pain during castration, cortisol levels up to 48 h, behaviours and postures, lesions, palpation, bodyweight measurements, histology	Active behaviour (especially the rubber ring lambs), scrotal swelling, palpation (9 days). Lesions were present >21 days.	[64]
Sheep, breed not specified	1 week vs. 4–6 weeks	Castration, rubber ring vs. combined Burdizzo/ring vs. sham handling	30 (6)	None stated	4 (castration day 2)	3	Moving (including play), eating, standing, lying and abnormal postures	Play behaviour, reduced lying, and abnormal posture	[65]
Sheep, breed not specified	45 days	Tail docking, cautery iron vs. sham handling	50 (25)	Lignocaine 2% 2 mL injected locally prior to docking)	90	90	Infra-red thermography, Mechanical nociceptive threshold, inflammation, histopathology	Mechanical nociceptive threshold, inflammation (significance to day 30), histopathology (moderate to marked fibrosis of the epineurial and perineurial connective tissue, nerve proliferation)	[66]
Sheep, Merino	10–12 weeks	Mulesing, Sodium lauryl sulfate (SLS) injection vs. surgical vs. sham handling	32 (10–11)	Topical local anaesthetic as a wound dressing for surgically mulesed group	42	7	Haematology, cortisol, haptoglobin, β-endorphin, rectal temperature, body weight, standing postures, ADG	ADG, haptoglobin	[67]
Sheep, Merino	6–7 months	Mulesing, surgical vs. Sham	20 (10)	None stated	113	112	Wound healing, Paddock observations of behaviour (lying, grazing), arena observations of handler aversion, cortisol and β-endorphin, growth rate	Wound healing (by day 22), handler aversion (up to day 112), weight gain (day 14)	[22]
Sheep, Merino	10–12 weeks	Mulesing, surgical vs. intradermal injection SLS vs. skin clip vs. none (control)	44 (11)	None	25	25	Plasma cortisol, haptoglobin, weight, gait	In surgical mulesing: decreased weight gain (to day 25), lower feed intake (to day 15), higher cortisol levels (to Day 7), higher haptoglobin (to day 14)	[68]

* The use of general anaesthesia that was reversed after the procedure is not included; ** Parameters indicating long term pain were experienced to the length of time in the “Duration of pain” column, unless otherwise specified.

**Table 2:** Analgesic and anti-inflammatory medications available for prescription in Australian Veterinary Practice [35,36,37,69,70].

Drug Type	Use	Schedule in Australia	Common Side Effects	Example Generic Molecules in this Class
Opioid	Analgesia, sedation, strong pain relief	8	Bradycardia, respiratory depression, sedation, constipation, tolerance	Methadone, butorphanol, buprenorphine, tramadol *, morphine *
NSAID	Analgesia & anti-inflammatory, chronic and acute	4, 5	Renal & hepatic toxicity, mild and transient vomiting, soft stool, inappetance, lethargy, gastrointestinal erosions/ulcerations	Meloxicam, ketoprofen, flunixin, tolfenamic acid, carprofen, grapiprant, other coxibs
Corticosteroid	Anti-inflammatory and immunosuppression	4	Hepatopathy, hyperlipidaemia, diabetes, delayed wound healing, immunosuppression leading to infection, GI ulceration. Use with NSAIDs can lead to increased risk of GI injury	Dexamethasone, prednisolone, prednisone
α_2_ Agonist	Sedation, muscle relaxation & analgesia	4	Profound sedation, vomiting, startle behaviour, bradycardia, respiratory depression, hypothermia	Clonidine, detomidine, dexmedetomidine, medetomidine, xylaxine
Local Anaesthetic	Pain blocking/prevention	4, 5	CNS stimulation in large doses	Lignocaine, procaine, bupivacaine, prilocaine, mepivacaine
Other therapies and off-label products	Sedation, potentiation, analgesia	Various	Sedation (except paracetamol)	Diazepam, gabapentin *, paracetamol ** cannabidiol *

* Not registered in Australia for animals but used in veterinary practice. Gabapentin off-label use is widespread and very common for the treatment of neuropathic pain as well as a sedative/anxiolytic in companion animals [70]. Cannabidiols are gaining traction as a pain relief option in companion animals [71] and may be scripted for various purposes, including chronic pain, under state-by-state regulations in Australia [72]. ** Paracetamol is only registered by the APVMA in Australia as an anti-pyretic in piglets [37], but is commonly used off-label for analgesia [69,73].

**Table 3:** Summary of registered Australian products with specific claims for surgical pain in cattle and sheep [37].

Product (Brand if Applicable)	Prescription or OTC	Drug Class	Duration of Action *	Claim (Associated with Surgical Pain)
Lignocaine 2%, Prilocaine 2% (cattle only)	Prescription	Local anaesthesia pre-procedure	1–4 h	Infiltration anaesthesia and nerve block
Bupivacaine 0.4%, lignocaine 4%, adrenaline, cetrimide (Tri-Solfen)	OTC	Local anaesthesia post-procedure	After 30 s and up to 4 h	Topical local anaesthesia and antiseptic spray for castration, mulesing and tail docking in lambs, and castration and dehorning or disbudding in calves.
Lignocaine 2% (sheep only) (Numocaine for Numnuts device)	OTC	Local anaesthesia peri-procedure	Up to 3 h	Local anaesthestic injection via Numnuts applicator for tail docking and castration via rubber rings in sheep
Meloxicam 0.5% injection (cattle only) 2% injection, 4% injection (cattle only)	Prescription	NSAID	No duration of action specified on the label	Cattle—to assist in the control of pain particularly that after heat cautery dehorning in young cattle. It is recommended that a cornual nerve block anaesthesia is used in conjunction for dehorning. Sheep: As a single dose for alleviation of pain and inflammation pain in sheep more than 14 days old.
Flunixin 5% (cattle only)	Prescription	NSAID	24–36 h	Suppression of post-operative swelling and lameness
Meloxicam 1% (Buccalgesic, Butec)	OTC	NSAID	No duration of action specified on the label	Oral Transmucosal NSAID for alleviation of pain in lambs after mulesing, tail docking and castration, and in conjunction with a cornual block in calves for disbudding and dehorning, and in conjunction with a local anaesthetic for castration to enhance pain relief and minimise tissue damage and distress.
Meloxicam 1.5% oral (Meloxi-care)	Prescription	NSAID	No duration of action specified on the label	For the reduction of pain and inflammation associated with band or surgical castration administer orally two hours before the painful procedure.

* Claimed on label.

## 3. Pain Duration after Routine Husbandry

To understand the length of time that pain is experienced after a surgical procedure, it is necessary to consider the physiological mechanism underlying the type of pain. Pain itself is a protective mechanism, as it signals for potential or actual tissue damage, and ensures that an animal (if able) moves away from or avoids further injury [74]. Pain that is induced by surgical procedures should and can be pre-empted and mitigated to an appropriate degree.

During the initial phase of an injury, nociceptors are activated, nerve fibres deliver the sensation of pain to the brain, and the response causes the body to flinch or move away from the pain source. The tissue damage at the site causes the release of inflammatory and other mediators, which initially activate the nociceptors, and persistent pain sensitises those nociceptors [75] leading to longer-term pain.

Inflammation at a local level is a tissue stress response by the body’s immune system, whereby damaged tissue, and infected or necrotic cells are identified and removed [76] and the healing process is initiated. The immune and vascular response of inflammation, which includes the formation and release of prostaglandins, involves (at a tissue level) redness, swelling, heat, and pain at the site of injury [77]. Damage from injury is detected by both tissue-resident macrophages and nociceptors at the injury site. Inflammatory mediators are responsible for inflammatory pain, while prostaglandins can enhance the sensitivity of nociceptors by lowering their threshold for activation, thus increasing the pain sensation [78]. While the inflammatory response is vital for healing [77], inflammatory pain can be intense and lead to an abnormally heightened sensitivity to pain (hyperalgesia), pain experienced from usually non-painful stimuli (allodynia), and sustained or increased pain perception (sympathetically maintained pain) [6].

The bulk of research performed to date regarding pain mitigation in livestock has focused on the acute, immediate pain experienced during a procedure and in the following 2–8 h. However, there have been several studies in animals showing that post-procedural pain lasts for longer than the first few hours, with neuropathic or inflammatory pain being postulated as the likely cause [79]. An example of this longer-lasting pain has been established after rubber ringing (ischaemic amputation) for tail docking and castration. The constrictive rubber ring leads to ischaemic necrosis of the tissue, which ultimately sloughs away, making the procedure bloodless but intensely painful, with significant behaviours indicative of severe pain, such as rolling, writhing and abnormal standing shown for at least 4 h after the ring is placed [80], then other observations such as reduced playing and lying, wound-directed behaviours and swelling, as well as atypical postures and abnormal walking seen for several days afterwards [52,81]. This is particularly interesting in the context of the Australian production industry, given that in recent Australian industry surveys of 2003 sheep producers and 803 beef producers, it was reported that 98% of male lambs and 85% of male calves owned by the producers surveyed are castrated with rubber rings, with only a quarter of these receiving any form of pain relief [16,17]. Studies in other procedures commonly performed, such as surgical castration, tail docking, dehorning and others have been shown to cause pain for days or weeks afterwards [40,46,53].

A selection of studies that collected pain data for more than 3 days, and that variously investigated aspects of routine husbandry methods in livestock is presented in Table 1; while many were not specifically designed to do so, the studies illustrate that observations of pain have been made for days or weeks following these procedures.

Pain behaviours have been observed for days or weeks post procedure, and many of the studies seen in Table 1 were still observing pain-related behaviour on the last day of recording. It is therefore possible that pain continued undocumented after these studies were completed, and this limits conclusions as to the true extent or duration of pain experienced. There are limited studies specifically designed to evaluate the duration of pain, but rather the focus of much research into pain relief has been intended to compare procedure methods, ages, or acute pain relief, so the assessments of pain in studies have not been specifically designed to detect longer lasting or inflammatory pain [42,43]. Some techniques used for measurement of pain (such as palpation or pressure) tend to induce pain, so that the animal may have been relatively pain-free without interference, thus confounding the interpretation of persistent pain. It is also of note that in the studies presented in Table 1, animals that were provided with acute pain relief still experienced observable pain for days and sometimes weeks afterwards [41,46,53].

These findings of longer-term post-procedural pain are not unexpected if considered in the context of the human experience. A human patient who has experienced amputation or major abdominal surgery is routinely provided with significant pain relief for several days post injury or surgery, since it is understood that surgery will cause the release of inflammatory mediators, which activate nociceptors, and if the post-surgical pain is persistent, the nociceptors become sensitised. Prolonged inflammatory states leading to this sensitisation can cause changes to the nociceptors that can lead to chronic pain pathophysiology [75]. Examples of this in humans who have undergone what may be considered equivalent surgeries have been reported, with one study [82] relating that up to 50% of patients who have had an amputation of a limb or digit will experience pain for at least 6 months, while in another study [83] 32% of hysterectomy patients still reported pain after 6 months. It is therefore highly likely that animals who have experienced amputation of tail or horns, or spaying/castration, may experience pain for a similar period.

If the inflammatory response (and therefore pain) is resolved during normal wound healing, the central nervous system (CNS) will revert to normal activity, thus avoiding long-term chronic pain caused by changes to the nociceptors via inflammation [75]. Extrapolation of this concept to non-human mammals demonstrates a need for pain mitigation in animals that decreases the inflammatory response for a longer period than the acute peri-or post-operative phase if long-term or chronic pain is to be avoided.

## 4. The Current State of Pain Mitigation

In animals, the medications available for pain relief or pain mitigation are limited, and the products available over the counter to owners are even fewer.

Different classes of drugs act in different ways upon the body, and the ideal analgesic targets the cause or mechanism of the pain [84], and that allows animals to achieve functionality and normal behaviour as soon as possible. This may mean that the method of pain relief changes throughout the injury and healing, or that multi-modal pain relief is required.

There are six broad categories of drugs, (Table 2), that are used in the treatment of pain and inflammation: opioids, non-steroidal anti-inflammatory drugs (NSAIDs), α_2_-agonists, local anaesthetics, corticosteroids, and “others”. These others such as non-opioid analgesics and antipyretics, or tranquilisers or anticonvulsants whose primary purpose may not be analgesia but may act as an adjunct to known analgesics.

Pain mitigation after non-routine or major surgery in animals is usually tailored to the specific animal and circumstance, and as per Table 2, there are several options available for appropriate multi-modal post-surgical pain relief.

The fact that most research has concentrated on the immediate pain associated with procedures is reflected in the current products registered in Australia for the mitigation of pain in cattle and sheep. These include a topical local anaesthetic (Tri-Solfen Wound Anaesthetic and Antiseptic Solution, Dechra Veterinary Products Ltd. (Somersby, NSW, Australia), specialised local anaesthetic in a device (for rubber ringing only) (Numnuts device with lignocaine), and oral trans-mucosal meloxicam (Butec OTM, Troy Laboratories Ltd., Glendenning, NSW, Australia) available over the counter, or injectable local anaesthetic or NSAIDs available from a veterinarian [37]. Other livestock species such as pigs and goats have fewer registered products available and generally require a veterinary prescription for off-label use. Local anaesthetic products are effective for approximately 1–4 h post procedure depending on the dose rate, molecule and/or combination used and NSAIDs, depending upon the product, will be effective for 4 to 36 h [36]. A summary of Australian products registered for post-husbandry pain relief in cattle and sheep is shown in Table 3.

When considering the context of routine livestock husbandry on the farm, or the conduct of minor surgical procedures in the clinic, the requirement for a universal approach to pain mitigation in a large number of animals accounts for several parameters in addition to efficacy: availability (over-the-counter vs. prescription), practicality (single dose), and ease of application for non-veterinarian users. Another consideration is the selection of an active pharmaceutical ingredient (API) that has an established safety and efficacy profile across various species, including the food safety aspect in production species, and that is economically viable. Finally, there is the requirement for the animal to be able to function normally whilst under treatment, which precludes many of the drugs that affect the central nervous system. One of the major signs of recovery in livestock is the ability to “mother up” and/or graze effectively, as well as move from the point of treatment to the paddock as soon as practicable after treatment.

The NSAID group of products meets many of the above requirements, and there are several NSAIDs registered in livestock in Australia that meet the efficacy and safety criteria, providing relief relatively quickly after the first dose whilst still allowing the animal to be ambulant and functional (Table 3). In their current form, however, they do not provide pain relief for an adequate period when considering the length of pain duration experienced by animals after procedures (Table 1). NSAIDs are known to have some general contraindications, most of which are relevant in an older or debilitated population; such as renal, hepatic, cardio or pulmonary insufficiencies or dysfunctions, animals that are pregnant, or animals on concomitant systemic corticosteroids or other NSAIDs, or that are dehydrated [37,85].

## 5. Future Directions: Longer-Term Pain Relief

The need for the provision of pain relief for an appropriate length of time in animals undergoing surgery or painful routine procedures is becoming increasingly recognised [86]. It is a recommendation that pain relief should be provided for at least 72 h post surgery [87], but with current registered products available to veterinarians and owners, retreatment at hourly or daily intervals is required to achieve this level of pain mitigation. When an animal is hospitalised post surgery, this can be easily achieved. However, most animals are discharged on the same day or within 24 h of surgery, and for livestock especially, which undergo routine procedures on-farm, the stress to the animals of re-handling (mustering, physical separation and restraint, the risk of re-injury from handling and restraint, and needle sticks) that would be needed to re-treat, can negatively affect the animal’s welfare. Another important consideration when using a product that provides sustained pain relief is the time to onset of action. An ideal product for long-acting pain relief is one which has rapid onset of analgesia, and then maintains this over a sustained period without requiring re-treatment.

One method of providing medication that does not require rehandling is in-feed medication, where the animals voluntarily consume nutritional supplements in the form of licks or blocks that contain the drug of interest, allowing the provision of medication over several days or even weeks, in a non-invasive manner. This has been reported for self-medication of endo parasiticides in wildlife and zoo animals [88], and sheep [89]. There is work underway with pain relief medication and voluntary consumption of NSAIDs through medicated feed or supplements has been trialled with carprofen in chickens with lameness [90,91] flunixin in cattle [92] and sheep [93,94] and meloxicam in cattle [95]. It has been found that this method provides an ongoing level of pain relief for animals and that future research in this area is warranted. Some of the challenges for livestock dosing include ensuring appropriate palatability so that voluntary consumption of adequate but not excessive medication is achievable, and managing accurate dose rates for medications of this type, especially considering the differences in pharmacokinetics of the oral route in monogastric versus ruminant species, as well as determining the withholding periods for meat and/or milk. In addition, maintenance of the drug product potency in this format where exposure to heat, moisture and UV radiation may destabilise the API needs to be evaluated.

The application of transdermal medication has long been an option for providing pain relief in humans, and a buprenorphine solution for cats delivered as a low volume topical dose to the unclipped dorsal cervical skin provides extended plasma buprenorphine concentrations and opioid physiological effects [96]. A study in cattle using transdermally-delivered ketoprofen as a back-line pour-on compared with conventional intramuscular administration [97] showed that the transdermal formulation was slightly superior in terms of overall drug exposure, giving rise to the possibility of transdermal delivery of longer-acting NSAIDs (such as meloxicam) providing a greater duration of action, although this concept is unproven. A study in sheep using transdermal patches of fentanyl [98] in order to decrease the need for post-operative handling showed promise, providing up to 72 h of pain relief; however, fentanyl as a drug product would not be feasible for large-scale use in livestock, given the practicality of an adherent patch on fleece or hair, which would need to be clipped or shorn to allow adherence to the skin, and the possibility of animals removing and ingesting the patch during self or social grooming leading to toxicity. In addition, the controlled scheduling that is used to prevent potential abuse and misuse, and consequent difficulty of procurement adds another layer of complexity. A recent and promising development in the delivery of transdermal pain relief is lignocaine-impregnated elastrator bands (developed by Chinook Contract Research Inc., Canada) that have been tested in calves and lambs [99,100,101], with results showing effective levels of lignocaine in tissue for 3 to 7 days, although the sloughing of the scrotum and testes tended to be slower when compared with conventional bands in a larger trial of lambs [102].

Another method of extending the pain relief available to animals, especially livestock where re-handling would exacerbate stress, is to develop an extended-release (ER) or sustained release (SR) pain relieving medication, in the form of an anaesthetic, analgesic or NSAID that is dosed once at the time of surgery.

There is currently a liposomal encapsulated bupivacaine injectable suspension under investigation for the extension of the duration of local anaesthesia, and in dogs undergoing cranial cruciate ligament rupture surgery, an intra-thecal injection of the sustained release bupivacaine at the time of wound closing has shown promising results with some pain relief still present up to 72 h after surgery [86]. In a subsequent study of dogs undergoing similar surgeries, the animals receiving liposomal encapsulated bupivacaine injection were less likely to require rescue analgesia and required lower amounts of opioids than the dogs that received conventional bupivacaine [103]. Another novel formulation of bupivacaine involving sucrose acetate isobutyrate, a highly viscous sugar that has also been used for the sustained release of drugs, has been tested as a cornual nerve block when disbudding calves. The level of anaesthesia was prolonged (8–36 h) when compared with a lignocaine cornual block (0.5–1.5 h) [104], or bupivacaine cornual block (4 h) [105].

A compounded sustained release formulation of injectable buprenorphine (an opioid analgesic) has been tested in sheep [106] and guinea pigs [107,108] and has been shown to provide a steady state of the minimum threshold for therapeutic benefit for 72 h in sheep and up to 48 h in guinea pigs. This may be an option for veterinarian use; however, it is not an option for livestock owners due to the scheduling constraints of buprenorphine, which is a strictly controlled drug in Australia and other countries.

From a practical standpoint, to allow owner-treatment of livestock, an NSAID provides a practical solution to longer-acting pain relief. A sustained release injectable formulation of meloxicam in a polymer-based matrix has been trialed in sheep [87], but the formulation provided only 48 to 60 h at a presumed therapeutic level of meloxicam and requires further investigation. Although studies have shown that pain from routine husbandry such as castration, tail docking and dehorning lasts for weeks to months [55,57,60,61], the main pain indicators that impede normal function such as walking, eating and socialization generally persist for at least 3 to 7 days [43,62,109,110]. A sustained-release formulation of a well-characterized drug product with a good safety profile such as meloxicam, which provided a therapeutic level of pain relief for 72 to 96 h, would substantially improve the welfare and productivity of animals that undergo routine husbandry on the farm.

A further option for longer-term pain relief in livestock is the use of some of the NSAID APIs that are currently registered in humans or companion animals only. The NSAID mavacoxib, a long-acting COX-2 inhibitor, has a half-life in dogs of more than 2 weeks [111], so investigation of its efficacy in livestock may lead to an efficacious and longer-acting pain relief product.

The safety concerns with regards to the use of NSAIDs are generally focused on animals with renal, hepatic, pulmonary or cardiac insufficiencies or dysfunction, with gastrointestinal disease, or that are being treated with concomitant systemic corticosteroids or NSAIDs [85]. For the target population species indicated for this project, the animals are young and generally in good health, and not being treated with other NSAIDs or steroids. One issue that must be considered is that in animals that have experienced trauma with active haemorrhage or blood loss, the use of NSAIDs is contraindicated [85], which may preclude certain procedures, such as mulesing, from being treated with some NSAID (especially those that include significant COX-1 inhibition) sustained release formulations.

## 6. Conclusions

As livestock handlers, owners and veterinarians become better at recognising situations where pain and distress are experienced, they should strive to improve methods of pain mitigation. Inflammatory pain post surgery is a well-established concept and demonstrates a requirement for mitigating the inflammatory response post surgery, ideally for at least 5 to 7 days.

All the NSAIDs currently available for use in veterinary practice in Australia provide relief from inflammatory pain and have been shown to meet the appropriate safety criteria in many species; however, current products require frequent retreatment to provide an adequate period of pain mitigation, posing practical difficulties, especially for livestock. The challenge is providing a solution that allows a single dose to provide relief for at least a week or longer, to animals that are only handled once (at the time of surgery), or for which repeated doses are not viable. Currently, there are no commercially available, registered anti-inflammatory solutions for livestock available in Australia or globally, that will provide an adequate level of pain mitigation for an extended period. Potential solutions being researched include in-feed dosing via voluntary consumption of medicated feed, transdermal medication delivery, and extended-release formulations. Continued research into the development of extended-release formulations for pain mitigation in livestock is warranted to provide better animal welfare now and in the future.

## Data Availability

No new data were created or analysed in this review; therefore, data sharing is not applicable.
